# Vitamin D: Pharmacokinetics and Safety When Used in Conjunction with the Pharmaceutical Drugs Used in Cancer Patients: A Systematic Review

**DOI:** 10.3390/cancers5010255

**Published:** 2013-03-11

**Authors:** Deborah A. Kennedy, Kieran Cooley, Becky Skidmore, Heidi Fritz, Tara Campbell, Dugald Seely

**Affiliations:** 1 Canadian College of Naturopathic Medicine, 1255 Sheppard Avenue East, Toronto, Ontario, M2K 1E2, Canada; E-Mails: dkennedy@ccnm.edu (D.A.K.); kcooley@ccnm.edu (K.C.); bskidmore@rogers.com (B.S.); hfritz@ccnm.edu (H.F.); ndtara@gmail.com (T.C.); 2 Ottawa Integrative Cancer Centre, 29 Bayswater Avenue, Ottawa, Ontario, K1Y 2E5, Canada

**Keywords:** vitamin D, calcitriol, pharmacokinetics, drug interactions, systematic review

## Abstract

Vitamin D has reported anti-cancer and anti-inflammatory properties modulated through gene transcription and non-genomic signaling cascades. The purpose of this review was to summarize the available research on interactions and pharmacokinetics between vitamin D and the pharmaceutical drugs used in patients with cancer. Hypercalcemia was the most frequently reported side effect that occurred in high dose calcitriol. The half-life of 25(OH)D_3_ and/or 1,25(OH)_2_D_3_ was found to be impacted by cimetidine; rosuvastatin; prednisone and possibly some chemotherapy drugs. No unusual adverse effects in cancer patients; beyond what is expected from high dose 1,25(OH)_2_D_3_ supplementation, were revealed through this review. While sufficient evidence is lacking, supplementation with 1,25(OH)_2_D_3_ during chemotherapy appears to have a low risk of interaction. Further interactions with vitamin D_3_ have not been studied.

## 1. Introduction

Vitamin D’s role and importance in bone metabolism has been known for many years. The influence of vitamin D status and the associated impact on health and disease represents yet another important potential role for vitamin D. Wang *et al*., in a recent meta-analysis on vitamin D status and the associated risk of cardiovascular disease (CVD) found a direct inverse association between circulating (25(OH)D_3_ levels and CVD risk to 60 nmol/L [1]. Further roles for vitamin D are also under exploration, such as its function in the immune system and providing resistance to infection, as well as its antiproliferative and anti-inflammatory activity [1,2,3,4,5].

The main source of vitamin D is through endogenous production in the skin. Vitamin D is synthesized by the action of UVB radiation activating the 7-dehydrocholesterol molecule in the skin and converting it to pre-vitamin Vitamin D_3_ (cholecalciferol). In this form, it is transported in the blood to the liver, bound to either albumin or vitamin D binding protein (DBP) [6]. In the liver, it is thought to be hydroxylated by 25-hydroxylase, a member of the CYP2R1 enzyme family, through specific enzyme(s) that still need to be elucidated, to 25-dehydroxyvitamin D_3_ [25(OH)D_3_, calcidiol] [7]. Serum levels of 25(OH)D_3_ are affected by vitamin D_3_ intake and production by the skin, as there is little regulation of the conversion of cholecalciferol to 25(OH)D_3_ within the liver [7]. From the liver, 25(OH)D_3_ is transported to the kidney, where again hydroxylation occurs, this time by the enzymatic action of 1α-hydroxylase, a member of the CYP27B1 family, to 1,25-dihydroxyvitamin D [1,25(OH)_2_D_3_, calcitriol] [7]. 1,25(OH)_2_D_3_ is catabolized by the action of 24-hydroxylase, a member of the CYP24A1 family, to calcitroic acid and excreted in bile. 25(OH)D_3_ is the major circulating form of vitamin D, while 1,25(OH)_2_D_3_ is the major active form of vitamin D. The liver and the kidney are the primary locations for conversion of vitamin D along its activation pathway; however, they are not the only locations where the conversion of vitamin D_3_ to 25(OH)D_3_ is possible [6,8].

Vitamin D_3_ is stored in the adipose tissues of the body and its half-life is approximately 2 days, while 25(OH)D_3_’s half-life is approximately 3 weeks [6]. When supplementation with vitamin D_3_ is in excess, adipose tissue can become saturated and Vitamin D_3_ readily converted to 25(OH)D_3_ [6]. It is believed that 25(OH)D_3_ is responsible for the toxicity of vitamin D since there are no known regulator mechanisms within the body for this conversion to 25(OH)D_3_ [6,9]. While, 1,25(OH)_2_D_3_ serum concentrations are tightly regulated through feedback mechanisms related to serum calcium and phosphorus concentrations and has a half-life of between 10–20 h [6,7]. In situations where vitamin D_3_ intake has been in excess, rarely, have there been correspondingly high 1,25(OH)_2_D_3_ levels, however, high intakes of calcitriol can override the feedback mechanisms [10].

Vitamin D’s role in the maintenance of bone mineralization is affected through the elevation of calcium and phosphorus in the blood at concentrations that result in mineralization of the skeleton [8]. In addition, the anti-cancer and anti-inflammatory effects of vitamin D are regulated through gene transcription via the vitamin D receptor (VDR) and through non-genomic signaling cascades [2]. Vitamin D acts to block the cell cycle and slow cellular growth, promote apoptosis, modulate angiogenesis and regulate prostaglandin metabolism and signaling [2]. Hence, these signals have led researchers to explore its use in cancer prevention and treatment through epidemiological studies and randomized controlled trials in cancer [11,12,13,14,15,16,17]. Further, *in vitro* studies suggest that vitamin D can act synergistically with several different chemotherapeutic agents [18], creating uncertainty as to if and how vitamin D supplementation might be incorporated into a chemotherapeutic regime for cancer patients.

The objective of this review is to summarize the available evidence on the interactions between vitamin D and pharmaceutical drugs used in patients with cancer including the impact of vitamin D on the pharmacokinetics of these drugs and also any changes in vitamin D pharmacokinetics due to the drugs themselves.

## 2. Results and Discussion

**Figure 1:**
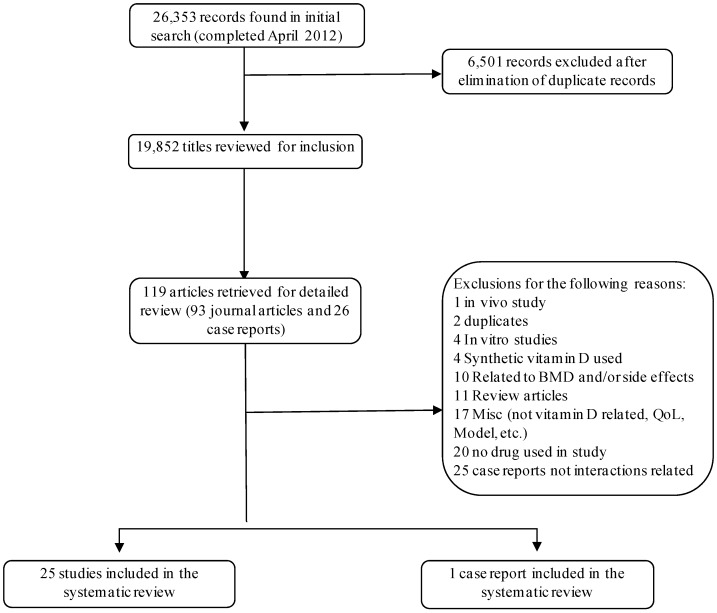
PRISMA search strategy flow chart.

There were 26,353 records reviewed for inclusion in the systematic review. After excluding duplicate records and screening based on title/abstract and then full text, twenty-six articles fitted the inclusion criteria. Figure 1 details the search strategy flow. The appendix contains the detailed search strategy for the OVID MEDLINE^®^ search. The majority of the papers found were in English, with one case report in French [19]. Details of the studies are summarized in Appendix Tables A1, Tables A2. There were a variety of different pharmaceutical drug and vitamin D combinations studied. Table 1 provides an overview of the various drugs included in the review and the form of vitamin D used in the studies. Cholecalciferol is the most frequently supplemented form of vitamin D, however, in the studies included in this review, calcitriol was the most commonly used form. Prostate cancer, in particular, and patients with solid tumors were the most well represented populations within the studies included in this review.

### 2.1. Overview of the Interactions

In general, there was no evidence found for positive or negative interactions between the drugs used in the treatment of cancer and vitamin D in cancer patients. Several studies report gastrointestinal cramps and ulcerations after the administration of high dose 1,25(OH)_2_D_3_ [20,21,22]. The use of calcifediol and thiazide medications in the elderly may present a cause for concern as one case report was found reporting hypercalcemia in two individuals [19].

Hypercalcemia, an expected side effect of high dose vitamin D therapy alone, was also the most frequently reported side effect that occurred in conjunction with the various pharmaceutical drugs included in the review.

*In vivo* studies have identified that a calcitriol peak plasma concentration of 10 nmol/L has significant anti-tumor activity [23,24]. In several studies, as a strategy to increase the serum vitamin D levels to parallel those peak plasma concentrations and AUC that, in vivo studies, suggested induced anti-tumor activity, dexamethasone was used to reduce the incidence of hypercalcemia at these higher doses of vitamin D [25]. The maximum tolerable dose (MTD) of calcitriol was found to be 74 µg/week; [23] however, with the addition of dexamethasone, the MTD was increased to 125 µg/week [26].

Hypophosphatemia was seen in two studies where docetaxel was used for prostate cancer but not in all studies that used this drug combination. In the Petrioli *et al*. study, 32 µg of calcitriol was administrated orally, once per week, in three divided doses, and most prostate cancer patients experienced hypophosphatemia [27]. While in the Tiffany *et al*. study, 60 µg of calcitriol was administered orally, once per week and 16.7% of the prostate cancer patients experienced hypophosphatemia [28].

In a case report, Boulard *et al*. reported elevated calcium levels, mental confusion, asthenia, constipation with fecal impaction with the use of calcifediol (vitamin D_2_) and thiazide medications in two elderly women over the age of 75 years [19]. All medications were halted and 45 mg/day of prednisone was administered; both cases resolved within one week. One of prednisone’s mechanisms of action is to reduce intestinal calcium absorption, and this seemed to help to resolve these women’s symptoms [29]. However, it is unclear as to the exact role of vitamin D in these cases, as some of these side effects reported are known risks of thiazide medications.

### 2.2. Impact on Pharmacokinetics

Several studies examined the pharmacokinetics of vitamin D during the course of treatment. calciferol was not found to impact the pharmacokinetics of gefitinib, or docetaxel [22,23,26]. Studies reporting on the pharmacokinetics are summarized in Appendix Tables A3.

Beer *et al.* evaluated the pharmacokinetics of 5 µg/kg of calcitriol by mouth (p.o.) and 36 mg/m^2^ of docetaxel intravenous (i.v.) alone, and in combination in five patients. They found no difference between the pharmacokinetics of calcitriol alone or with docetaxel [22]. The pharmacokinetics of orally administered calcitriol, in escalating doses, in combination with paclitaxel is presented in Appendix Tables A4 [30]. The pharmacokinetics were determined as part of a maximum tolerable dose finding study; which was halted when evidence of a reduction in calcitriol oral bioavailability become evident at the higher dose.

**Table 1:** Summary of pharmaceutical drug and vitamin D combinations included in the review.

Pharmaceutical drug	Calcitriol/DN-101	Calciferol	1α-Hydroxyvitamin D3
1,3-bis 1 nitrosurea		1	
13-cis retinoic acid			1
Altizide + spironolactone		1	
Carboplatin	1		
Cyclophosphamide		1	
Cytarabine	1		
Cytosine			1
Cytosine arabinoside			1
Dexamethasone	7		
Docetaxel	7		
Estramustine	1		
Gefitinib	2		
Hydrochlorothiazide		1	
Interferon		1	1
Melphalan		1	
Mitoxantrone	1		
Naproxen	1		
Paclitaxel	1		
Prednisone	1	1	
Zoledronate	1		

Studies on the pharmacokinetics of iv administered 1,25(OH)_2_D_3_ were also identified. Appendix Tables A4 summarizes the pharmacokinetics of i.v. administered calcitriol in increasing doses from two different studies [23,26]. Fakih *et al*. found that gefitinib did not have any impact on calcitriol pharmacokinetics; this finding was also confirmed in a pharmacokinetic study conducted by Muindi *et al*. [23,26], who compared the serum calcitriol plus dexamethasone concentration *versus* time plots from this study with the calcitriol only results from the Fakih *et al*. study [23], and reported that dexamethasone had no impact on calcitriol PKs [26].

Several studies reported on the impact that pharmaceutical drugs had on vitamin D metabolism. Investigations into the impact of statin medication on vitamin D metabolism were conducted in two studies. Rosuvastatin was found to increase both serum 25(OH)D_3_ and 1,25(OH)_2_D_3_ levels in both studies [31,32]. This may not be a drug class effect, since fluvastatin did not have the same impact on serum vitamin D parameters [32].

Odes and colleagues investigated the impact of cimetidine on vitamin D metabolism in nine participants during the spring months where there was increasing sun exposure [33]. They found that the anticipated increase in serum 25(OH)D3 levels from the increased sunlight did not occur in these individuals. There was no impact on 24,25(OH)D_3_ nor 1,25(OH)_2_D_3_.

A small study investigated the impact of prednisone on vitamin D metabolism in four healthy subjects. Avioli *et al*. found that 30 mg/day of prednisone altered vitamin D metabolism, reducing the half-life of 25(OH)D_3_ by 40–60%, and also reduced the vitamin D metabolite responsible for intestinal calcium absorption [29]. Briefly in this study, the pharmacokinetics of 1,2^3^H-vitamin D_3_ were established in four normal healthy adult volunteers over a five day period. Ten µCi of radiochemically pure 1,2^3^H-vitamin D_3_ was administered orally after a 16 h fast and blood samples were obtained at 5, 15, 30 and 45 min and at 1, 2, 4, 8, 12, 16 and 24 h for the first 24 h period and then every 12 h for an additional four days. After a two week wash-out period, each volunteer was given 30 mg of prednisone for 10 days. On Day 5, 1,2^3^H-vitamin D_3_ was once again administered and blood samples were obtained according to the previously described schedule for the remaining five days of the study.

A small study involving four patients with gynecological malignancies examined the vitamin D metabolites before, during and after various chemotherapy regimens that included cisplatin. They found that, while there was variation in 24,25(OH)D_3_ and 25(OH)D_3_ levels during the study, 1,25(OH)_2_D_3_ levels were significantly reduced by the chemotherapy [34]. The authors suggested that the reduction in 1,25(OH)D_3_ levels may be attributed to cisplatin’s nephrotoxic profile that results in the kidney’s reduced ability to convert 25(OH)D_3_ to 1,25(OH)_2_D_3_.

A second study compared the change in serum vitamin D metabolites between colorectal cancer patients undergoing chemotherapy *versus* those who were not receiving chemotherapy through a retrospective chart review. The study found that patients receiving chemotherapy were more likely to have lowered 25(OH)D_3_ levels than those not receiving chemotherapy [35]. A recently published paper found the same result, lower serum 25(OH)D_3_ levels in breast cancer patients during and after chemotherapy treatment [36].

Our review found that high dose calcitriol used in conjunction with several different pharmaceutical drugs used in patients with cancer did not result in adverse events beyond what could be expected from the use of high dose vitamin D alone, *i.e*., hypercalcemia. There were, however, several areas identified that warrant further investigation.

Dexamethasone was used to reduce the incidence of hypercalcemia and achieve a higher maximally tolerated dose (MTD) in prostate cancer patients, while prednisone was used to manage the side effects of hypercalcemia [19,26]. Both medications act by reducing intestinal absorption of calcium as a means to reduce the hypercalcemic state [19,26]. Avoili *et al*. found that prednisone reduced the half-life of 25(OH)D_3_ whereas dexamethasone had no impact on 1,25(OH)_2_D_3_ levels [26,29]. Work in animal models suggests that dexamethasone may impact 1,25(OH)_2_D_3_ levels through up regulation of CYP24A1 transcription resulting in increased catabolism of 1,25(OH)_2_D_3_, indicating that more research into dexamethasone’s potential impact on vitamin D metabolism is warranted given its wide spread use in cancer patients and similar mechanism of action to prednisone [37]. Further, there is evidence that many cancer tumor types: breast, lung, colon and cervical as examples, over expresses CYP24A1 mRNA which may result in increased catabolism of vitamin D [37]. Several studies pointed to a reduction in serum 25(OH) D_3_ levels in patients undergoing chemotherapy when exposed to a broad base of different chemotherapy drugs [34,35,36]. Whether this is as a result of alterations in lifestyle from undergoing cancer treatments or from the chemotherapy drugs themselves is not entirely clear. Cisplatin does induce nephrotoxicity and reduced vitamin D levels have been reported in patients exposed to cisplatin. However, other chemotherapy drugs that do not induce nephrotoxicity have also induced these phenomena [34,35,36]. Vitamin D’s role in immune system modulation, potential chemopreventative role, and in bone metabolism suggests that monitoring of vitamin D serum levels during the course of treatment for cancer may be important in this vulnerable population.

While the search parameters of this review did not directly reveal an impact of vitamin D on P450 system nor drugs that have an impact on vitamin D levels through alternation of the enzymatic activity through the P450 system, there are several drugs that are known to impact vitamin D levels. The azole class of antifungal drugs, such as ketoconazole, flucinazole have been shown to inhibit the activity of CYP24A1 [37]. Genistein, a plant isoflavone found in *Glycine max* (soybeans) and in other plant products, has been shown, *in vitro*, to in inhibit transcription of both the CYP24A1 and CYP27B1 genes [37,38]. Work by Wang and colleagues have demonstrated that 1,25(OH)2D_3_ can be catabolized by a CYP3A4 dependent pathway, which is inducible by rifampin [39]. This CYP3A4 pathway maybe responsible for the osteomalacia-inducing aspects of several pharmaceutical drugs [37,39].

This review not only demonstrates the minimal evidence amassed relating to direct correlations between vitamin D and pharmaceutical agents employed in people with cancer, but also the limited collection of evidence relating to vitamin D metabolism in situations where normal organ or physiological function may be compromised due to pharmaceutical agents. 

A limitation of this review is that the studies were all conducted in small patient populations, limiting both the power and the generalizability of the results. Despite the rigorous design of some of the existing studies, more robust studies with larger sample sizes might help address individual variations which may impact vitamin D and chemotherapeutic regimes. This relative lack of research points to opportunities for further exploration on the impact of pharmaceutical drugs on vitamin D metabolism.

## 3. Experimental Section

### 3.1. Sources

Using an iterative process, a sensitive search strategy was developed and executed using the OVID platform. We searched OVID MEDLINE^®^ (1948 to March Week 4, 2012), OVID MEDLINE^®^ In-Process & Other Non-Indexed Citations (April 10 2012), Embase (1980 to 2012 Week 14), and AMED (Allied and Complementary Medicine, all years to 11 April 2012). The search employed both controlled subject headings (e.g., vitamin D, Cholecalciferol, Cytochrome p-450 enzyme system) and text words (e.g., vitamin D3, Calcitrol, CYP). The drug formulary of Cancer Care Ontario was used to identify cancer-related drugs, the names of which were also incorporated into the search. When appropriate, floating subheadings for concepts such as adverse drug reaction, drug interaction, and drug toxicity were included in the search strategy. We also searched the Cochrane Library on Wiley (including CENTRAL, Cochrane Database of Systematic Reviews, DARE, HTA, and NHS EED). No language or study group limits were applied to any of the searches. However, where possible, results were restricted to the human population. Previous reviews were hand-searched to identify other potentially relevant publications. A search of the WHO International Clinical Trials [40] and the MetaRegister of Controlled Trials databases [41] were also conducted to ensure that all relevant publications had been identified.

The strategy was peer reviewed prior to execution by an experienced information specialist using the PRESS Checklist [42].

### 3.2. Study Selection

#### 3.2.1. Inclusion Criteria

We selected all human studies and case reports using any form of vitamin D and a pharmaceutical drug used in patients with cancer. Studies were also included if they reported on the impact of vitamin D metabolism during the use of a pharmaceutical drug. No restrictions were placed on language of publication or country of study. The search results were imported into a bibliographic management tool (Thomson Reuters EndNote, Version X3, San Francisco, CA, USA). All titles were first reviewed to determine which articles to examine in greater detail. 

#### 3.2.2. Exclusion Criteria

Studies were excluded if a pharmaceutical drug was not included in the study; the study related to monitoring vitamin D levels in cancer patients as a means to manage side effects of medications; or a synthetic analog of vitamin D was used the study.

### 3.3. Data Extraction

Data extraction was carried out by one reviewer and independently checked for accuracy by a second reviewer. Data collected included the study location, year of publication, type of cancer, study design, participant numbers, drugs and dosage, form of vitamin D used, endpoints, study protocol and relevant reported outcomes. Additional information for pharmacokinetics studies was extracted and included: number of observations, dose, route of administration, half-life, C_max_, Area under the Curve (AUC_0–24__and_ AUC_0–72_) and clearance.

## 4. Conclusions

Of the hundred or so pharmaceutical drugs that are used in the treatment of cancer patients only a handful of these drugs have been studied in combination with vitamin D, primarily calcitriol (1,25(OH)_2_D_3_). The impact if any, of supplementation with vitamin D3 has not been reported on. It is reassuring to note that no unusual adverse effects in cancer patients, beyond what is expected from high dose 1,25(OH)_2_D_3_ supplementation, were revealed through this review.

Perhaps one of the most interesting findings from this review is that certain chemotherapeutic regimens appear to reduce serum 25(OH)D_3_ and/or 1,25 (OH)_2_ D_3_ levels during administration. This potential depletion combined with a lack of evidence for both pharmacodynamic and kinetic interactions suggests the need to monitor vitamin D levels during active cancer therapy and perhaps supplement with this agent during chemotherapy. Further research in this area is indicated as vitamin D status may have implications on the efficacy of conventional therapy for people living with cancer.

## Appendix

**Table A1:** Summary of the studies investigating vitamin D and pharmaceutical drugs used in the treatment of cancer patients.

Study/Year	Cancer type	Drug(s)	Form of Vitamin D	Design Treated/ Control	Study Endpoint	Study Protocol	Outcome
Cohen *et al.* [20]	Multiple myeloma	**BCP**: 1-3-bis (2-chloro-ethyl) 1-nitrosourea, cyclophosphamide and prednisone	Calciferol	RCT/cross over	(1) Compare directly the BCP regimen with MP	Pt randomized separately to either:	Toxicity of the supplemental drug package did not appear to be of major significance, there was greater GI toxicity for the active regimen.
					(2) Determine the response of patients initially resistant to one regimen when subsequently treated with the other;	BCP: BCNU, 75 mg/m^2^ i.v., and cyclophosphamide, 400 mg/m^2^ i.v., each in single doses, plus prednisone for 75 mg/day p.o. × 7 days; or MP: melphalan, 8 mg/m^2^/day p.o., for 4 days, and prednisone, 75 mg/day p.o., for 7 days. Each regimen was given every 4 week for 6 courses.	
		**MP**: melphalan and prednisone		373/0	(3) Determine if the combination of sodium fluoride, calcium gluconate, vitamin D, and fluoxymesterone could produce useful clinical benefit by repairing or strengthening bone structure.	Based on response to the above treatment, patients were then randomized to receive the active drug package (fluoxymesterone, 25 mg/m^2^ daily, sodium fluoride, 150 mg/day, calcium gluconate, 2 g t.i.d., and vitamin D (Calciferol), 50,000 U tabs twice a week, all given orally (p.o.), or a placebo package.	There was no significant difference between patients receiving the placebo package *vs.* the active agents in terms of bone pain, tenderness, or development of new fractures.
Hellstrom *et al.* [43]	Acute leukemia myelo-dysplastic syndromes	Cytosine arabinoside (Ara-c)	1α-hydroxy-vitamin D3	RCT	Study the efficacy and toxicity of each combination	1ST arm: IFN 3 million units per day, 13-*cis*-RA 1 mg/kg po per day, D3 start with 1 µg per day, increasing dose until mild hypercalcemia develops.	High rate of side effects due to IFN
		Alpha interferon (IFN)					
		13-*cis*-retinoic acid (13-*cis-*RA)		16/37/28/7		2ND arm: Ara-c 15 mg/m^2^ per day, if no pt response, increased to 25 mg/m^2^. 3RD arm: all four drugs given simultaneously	13-*cis*-RA and D3 were well tolerated and s/e (hypercalcemia) easily controlled with dose adjustment.
Slapak *et al.* [44]	AML	Cytarabine	Calcitriol	Uncontrolled study	Treatment of patients with AML over the age of 65 years.	Cytarabine was administered by continuous intravenous infusion at a dose of 20 mg/m^2^/day for 21 days.	Thirteen patients (45%) obtained a complete remission, and 10 patients (34%) had a partial response for an overall 79% response rate. There were three early deaths. The median remission duration was 9.8 months.
						Hydroxyurea 500 mg orally (po) q12 h was instituted 24 h prior to cytarabine and continued through day 21.	
		Hydroxyurea		28/0		Calcitriol (0.25 pg, PO Q12 h) was begun on the first day of cytarabine therapy and continued until relapse or the patient went off study	Two patients experienced hypercalcemia, in one patient calcitriol was held until normal levels were reached.
Muindi *et al.* [30]	Advanced solid tumors	Paclitaxel	Calcitriol	Phase I/PK	Determine the maximum tolerated dose and pharmacokinetics of calcitriol when administered with paclitaxel.	Escalating doses of calcitriol were given orally for 3 consecutive days each week, and paclitaxel (80 mg/m^2^) was given intravenously weekly.	No dose-limiting toxicity occurred in this trial. very high doses of calcitriol can be safely administered with paclitaxel. At a dose of 38 week no clinically significant hypercalcemia occurred.
						The starting dose of calcitriol was 4 µg and the maximum dose administered was 38 µg.	
				36/0		However, the study was halted since the study found decreased bioavailability of calcitriol with high dose oral administration.Dose escalation: 4, 6, 8, 11, 13, 17, 22, 29, 38 µg/day	
Beer *et al.* [45]	AIPC	Docetaxel	Calcitriol	Phase I/PK	Determine the safety and efficacy of weekly high-dose oral calcitriol and docetaxel.	Day 1: oral calcitriol (0.5_g/kg)	No obvious increase was seen in toxicity compared with phase II trials of docetaxel alone, with the possible exception of a somewhat higher than expected incidence of gastric and duodenal ulceration. PSA and measurable disease response rates as well as time to progression and survival are promising when compared with contemporary phase II studies of single-agent docetaxel in AIPC.
						Day 2: iv docetaxel (36 mg/m^2^)	
						repeated weekly for 6 weeks of an 8-week cycle.	
				37/0		Premedication with dexamethasone 8 mg orally 12 h and 1 h before docetaxel infusion and 12 after docetaxel infusion was given.	
Beer *et al.* [46]	AIPC	Carboplatin	Calcitriol	Uncontrolled trial	PSA response defined as a 50% reduction confirmed 4 weeks later.	Day 1: oral calcitriol (0.5 µg/kg) on day 1	Treatment-related toxicity was mild and generally similar to that expected with single-agent carboplatin. The addition of oral calcitriol to carboplatin in this study was not associated with an increase in the response rate when compared with the reported activity of carboplatin alone.
						Day 2: iv carboplatin (AUC 7 or AUC 6 in patients with prior radiation).	
				17/0		Repeated every 4 weeks.	
Morris *et al.* [47]	Progressive prostate cancer	Zoledronate	Calcitriol	Phase I	Examine the toxicity of pulse-dosed calcitriol with zoledronate and with the addition of dexamethasone at the time of disease progression	Calcitriol was administered for 3 consecutive days per week, starting at a dose of 4 µg per day. D	Calcitriol was well tolerated at doses up to and including 30 µg 3 times per week Peak plasma levels in the 24 µg and 30 µg cohorts were greater than the levels associated with antitumor effects preclinically.
						Doses were escalated to 30 µg per day.	
						Intravenous zoledronate (4 mg) was administered monthly. At doses above 6 µg/day.	
		Dexamethasone		31/0		examethasone could be added to the regimen at disease progression.	
Tiffany *et al.* [28]	AIPC	Docetaxel	Calcitriol	Phase I/II	Determine the safety and preliminary efficacy of the combination of high dose pulse calcitriol with a standard regimen of docetaxel plus estramustine.	Day 1: 60 µg calcitriol orally, and 8 mg dexamethasone bid for 1st 3 daysCycle repeated every 21 days for up to 12 cycles.	Treatment related grades 3 or greater toxicity seen in more than one patient included hypophosphatemia in 16.7% and neutropenia in 12.5%. Four patients had thromboembolic complications. High dose calcitriol may be safely added to docetaxel and estramustine administered on a 21-day schedule.
						Day 2: 60 mg/m2 docetaxel on day 2 (70 mg/m2 after cycle 1)	
						Day 1–5: 280 mg estramustine orally 3 times daily	
						Cycle repeated every 21 days for up to 12 cycles.	
		Estramustine		24/0		Patients also received 325 mg aspirin and 1 or 2 mg warfarin orally daily.	
Trump *et al.* [48]	AIPC	Dexamethasone	Calcitriol	Phase II	Evaluate high-dose calcitriol at a dose of 12 µg daily given X 3 plus dexamethasone weekly.	Oral calcitriol was administered weekly, Monday, Tuesday, and Wednesday (MTW), at a dose of 8 µg, for 1 month,	Toxicity was minimal: urinary tract stones in 2 patients; and a readily reversible, CTC (v.3.0) Grade 2 creatinine increase in 4 patients. The response rate reported in the current study (19%) was not found to be clearly higher than expected with dexamethasone alone. High-dose intermittent calcitriol plus dexamethasone appears to be safe, feasible, and has antitumor activity.
						at a dose of 10 µg every MTW for 1 month,	
						and at a dose of 12 µg every MTW thereafter.	
				43/0		Dexamethasone at a dose of 4 mg was administered each Sunday, and MTW weekly.	
Petrioli *et al.* [27]	HRPC	Docetaxel	Calcitriol	26/0	Evaluate the activity and tolerability of weekly high-dose calcitriol and docetaxel in patients with metastatic hormone-refractory prostate cancer (HRPC) previously exposed to Docetaxel.	Day 1: The treatment consisted of calcitriol (32 µg) given orally in three divided doses.	Most patients showed hypophosphatemia. No grade 4 toxicity or CHF. Weekly high-dose calcitriol and docetaxel seems to be an effective and well-tolerated treatment option for patients with metastatic HRPC previously exposed to docetaxel. High dose calcitriol seems to restore the sensitivity to the drug in patients who had progressed after an initial response to docetaxel-based chemotherapy.
						Day 2: iv Docetaxel (30 mg/m^2^) with dexamethasone 8 mg orally 12 h before, at the time of, and 12 h after docetaxel administration.	
						Administered on a schedule of six consecutive weekly administrations, followed by a 2-week rest interval.	
Fakih *et al.* [23]	Advanced solid tumors	Gefitinib	Calcitriol	Phase I/PK/PD	Determine the maximum tolerated dose (MTD) of this combination	Calcitriol was given i.v. over 1 h on weeks 1, 3, and weekly thereafter.	High doses of weekly i.v. calcitriol can be administered safely in combination with Gefitinib. Calcitriol concentrations achieved at the MTD 74 µg/week calcitriol exceed *in vivo* concentrations associated with antitumor activity in preclinical models. Dose-limiting hypercalcemia was noted in two of four patients receiving 96 µg/week of calcitriol. One of seven patients developed dose-limiting hypercalcemia at the MTD 74 µg/week calcitriol dose level
						Gefitinib was given at a fixed oral daily dose of 250 mg starting at week 2 (day 8).	
				36/0		Dose escalation: 10, 15, 20, 26, 24, 44, 57, 74, and 96 µg/week	
Beer *et al.* [49],	AIPC	Docetaxel	Calcitriol (DN-101)	RCT	The primary end point was prostate-specific antigen (PSA) response within 6 months of enrollment, defined as a 50% reduction confirmed at least 4 weeks later.	Weekly: docetaxel 36 mg/m^2^ intravenously for 3 weeks of a 4-week cycle combined with either 45 µg DN-101 or placebo taken orally 1 day before docetaxel. dexamethasone (4 mg orally 12 h before, 1 before, and 12 h after docetaxel administration).	Addition of weekly DN-101 did not increase the toxicity of weekly docetaxel. There were fewer gastrointestinal (2.4% *vs.* 9.6%; *p* = 0.02) and thromboembolic (1.6% *vs.* 7.2%; *p* = 0.03) serious adverse events in the DN-101 arm as compared with the placebo arm. All other categories of serious adverse events were balanced between the two groups.
						This regimen was administered weekly for 3 consecutive weeks of a 4-week cycle.	
				125/125		Primary hormonal therapy with gonadotropin-releasing hormone agonists or antagonists was maintained during the study.	
Chan *et al.* [50]	AIPC	Mitoxantrone	Calcitriol (DN-101)	Phase II	Evaluate the efficacy, safety, and impact on quality of life (QoL) of high dose calcitriol (DN-101) combined with mitoxantrone and glucocorticoids in androgen-independent prostate cancer (AIPC).	Day 1: 180 µg po of DN-101	DN-101 given every 3 weeks does not add significant activity to mitoxantrone and prednisone. The addition of DN-101 does not appear to increase the toxicity of mitoxantrone.
						Day 2: iv 12 mg/m^2^ mitoxantrone	
				19/0		Every 21 days with daily 10 mg po prednisone	
Beer *et al.* [51]	AIPC	Docetaxel	Calcitriol	RCT	Examine outcomes with intermittent chemotherapy in a large multi-institutional trial.	Day 1: calcitriol, oral dose of 45 µg or placebo	Increased duration of chemotherapy holidays, of the patients that took chemotherapy holidays, a substantial majority of evaluable patients (90.9%) retained their sensitivity to chemotherapy. There was no data on adverse events reported.
		Dexamethasone		45 of 250 patients participated in intermittent chemotherapy. Approximately 20% of patients treated with high dose calcitriol and 16% of placebo-treated patients received intermittent chemotherapy.		Day 2: docetaxel, iv. dose 36 mg/m^2^ with dexamethasone (4 mg orally given 12 h before, 1 before, and 12 h after docetaxel administration).	
		Placebo				This regimen was administered weekly for 3 consecutive weeks of a 4-week cycle.	
Muindi *et al.* [26]	Solid tumors	Dexamethasone	Calcitriol	Phase 1 & PK	MTD of weekly iv calcitriol with Gefitinib at 250 mg/day and dexamethasone 4 mg q12 h × 3.	Week1: 4 mg dexamethasone and Iv calcitriol	Combination was not associated with any clinical activity. Hypercalcemia occurred at all dose levels with increasing frequency and severity with higher DLs. MTD was 125 µg/week with co-administration of dexamethasone. This dose associated with consistent with calcitriol PK parameters associated with anti-tumor activity.
						Week 2: 250 mg Gefitinib daily	
						Week3: 4 mg dexamethasone, iv calcitriol with 250 mg Gefitinib daily.	
		Gefitinib		20/0		Escalating doses of calcitriol:57, 74, 96, 125, 163 µg/week	
Blanke *et al.* [52]	Pancreatic	Docetaxel	Calcitriol	Phase II	Determine time-to-progression for patients given this combination	Day 1: 0.5 µg/kg calcitriol p.o.	Hyperglycemia was attributed to dexamethasone. No significant hypercalcemia or myelosuppression seen. No significant toxicities attributable to calcitriol Results not superior to current therapy.
						Day 2: 36 mg/m^2^ DOX iv. + DEXA 4 mg orally given 12 h before, 1 h before, and 12 h after DOX administration).	
		Dexamethasone		25/0		Weekly for 3 weeks, then 1 week break.	
Srinivas and Feldman [21]	Prostate	Naproxen	Calcitriol (DN-101)	Single arm, open label Phase II	Determine whether the PSADT was prolonged. Secondary endpoints included: PSA response, defined as the first evidence of a total serum PSA decline of >50% from baseline maintained for at least 28 days and confirmed with two consecutive measurements taken two weeks apart; and duration of sustained response, defined as time from PSA decrease of >50% from baseline to the first evidence of disease progression.	Calcitriol (DN101): 45 µg once per week	The trial was halted after 21 patients were enrolled when a national trial comparing DN101 in combination with weekly docetaxel had a higher death rate in the DN101 arm compared to the new standard docetaxel dosing arm (every 3 weeks) and DN101 use was suspended pending further evaluation.
				21/0		Naproxen: 375 mg twice a day	These findings indicate that the combination of very high dose (45 µg) of weekly calcitriol (DN101) with daily naproxen (375 mg twice daily) was well tolerated in most patients. 3 patients developed severe abdominal cramps on the day following the DN101 dosing. The temporal relationship suggests that combination therapy may cause cramps in some patients, perhaps because of peak prostaglandin suppression at that time-point.
Chadha *et al.* [53]	CRPC	Dexamethasone	Calcitriol	Phase II	Response rate of iv calcitriol plus dexamethasone in CRPC pts.	Weekly treatment cycle:	Study was terminated for due to lack of patient response
						Day 1: 4 mg dexamethasone	
				18/0	Evaluate toxicity of high-dose iv calcitriol and dexamethasone in patients with CRPC	Day 2: 4 mg dexamethasone, the within 4–8 h later 74 µg calcitriol	Only one episode of grade ¾ toxicity (hypercalcemia) could be related definitely to calcitriol. Hyperglycemia > grade 2 was attributed to dexamethasone.
Scher *et al.* [54]	CRPC	Docetaxel	Calcitriol (DN-101)	Phase III/RCT	Compare survival times between weekly DOX+ DN-101 *vs.* every 3-week DOX + prednisone.	Control: 21-day dosing cycle with 5 mg oral prednisone bid, iv 75 mg/m^2^ on day 2, and 8 mg dexamethasone 12, 3 and 1 h prior to DOX infusion.	Study halted due to higher death rate in treated *vs*. control
						Treated: 28-day dosing cycle of 45 µg oral DN-101 on days 1,8 and 15	
				476/477	The comparative safety and tolerability was assessed by rates of AEs, grade 3, 4, and 5 AEs, SAEs and gastrointestinal events.	36 mg/m^2^ DOX days 2, 9, 16 and 8 mg dexamethasone 12, 3 and 1 h prior to DOX infusion.	Toxicity and number of dose modifications due to DOX were higher on the treated arm. No significant increase in severe DN-101 related AEs were observed.

AE: Adverse events; AIPC: Androgen-independent prostate cancer; AML: Acute Mylocytic anemia; Ara-c: Cytosine arabinoside; AUC: Area under the curve; BCP: 1-3-bis (2-chboroethyl) 1-nitrosourea, cyclophosphamide & prednisone; Bid: Two times per day; CHF: Congestive heart failure; CRPC: Castration- resistance prostate cancer; CTC: Common Toxicity Criteria; D3: 1 α hydroxyvitamin D3; DEXA: Dexamethasone; DL: Dose level; DN-101: a more concentrated caplet form of calcitriol that was produced by Novacea Inc.; DOX: Docetaxel; GI: Gastrointestinal; HRPC: hormone-refractory prostate cancer; IFN: Interferon; IV: Intravenous administration; H: hour; Kg: Kilogram; m^2^: Metres squared; µg: Microgram; mg: Millegram; MP: Melphalan and prednisone; pg: Pico grams; po: by mouth; PSA: Prostate specific antigen; PSADT: Prostate specific antigen doubling time; Q12 h: Every 12 h; QoL: Quality of life; RA: Retinoic acid; RCT: Randomized control trial; S/e: Side effect; SAE: Severe adverse events; Tid: Three times per day; U: Unit; *vs.*: *Versus*.

**Table A2:** Summary of the studies that report on vitamin D pharmacokinetics.

Study/Year	Participants	Drug(s)	DesignTreated/Control	Study Endpoint	Study Protocol	Outcome
Avioli *et al.* [29]	Healthy subjects	Prednisone	PK study	Demonstrate that the administration of prednisone leads to alternation in vitamin D metabolism and intestinal absorption of calcium.	**Day 1–14:** participants consumed a diet with 800 IU vitamin D.	Prednisone administration was associated with an abnormally rapid plasma turnover of Vitamin D, a decrease in the formation of a biologically active vitamin D metabolite responsible for effectively promoting calcium absorption from the intestines, and an overall decrease in the formation of the potent biologically active vitamin D metabolites. The half life of vitamin D3-3H was reduced by 40–60% after the administration of prednisone
					**Day 15:** participants took 10 µCi of radiochemically pure 1,2-3H-vitamin D. blood samples were taken at 5,15,30 45 min and at 1,2,4,8,12,16 and 24 h.	
					**Day 16–19:** blood samples obtained every 12 h.	
					**Day 20:** participants received 30 mg/day of prednisone for 10 days.	
			4 participants		**Day 25:** Day 15–19’s procedure was repeated.	
Odes *et al.* [33]	Patients with peptic ulcers	Cimetidine	Uncontrolled open label	Examine the effects if cimetidine on vitamin D hydroxylation in humans.	During spring months	Impact on vitamin D metabolites:
					**Dose:** 400 mg cimetidine bid for 4 weeks	
			9 participants		**Labs:** 25 hydroxyvitamin-D, 24,25-dihydroxyvitamin D, 1,25-dihydroxyvitamin D, calcium, phosphorus, potassium, urea, creatinine, uric acid, bilirubin, albumin, globulin, SGOT, SGPT and alkaline phosphatase were obtained at the baseline, at 4 weeks and 4 after the completion of treatment.	Prevented expected serum rise in serum concentration of 25 hydroxyvitamin-D. Levels of 24,25-dihydroxyvitamin D and 1,25-dihydroxyvitamin D were not affected
Gao *et al.* [34]	Gynecological malignancies	Various chemotherapy regimens	Uncontrolled open label	Examine the serially changes in vitamin D metabolites before, during and after chemotherapy.	Each person had a different chemotherapy regimen. Combinations of the following drugs:	Levels of 24,25-dihydroxyvitamin D and 25-dihydroxyvitamin D did not change consistently during the study. 1,25-dihydroxyvitamin D levels were significantly affected by the chemotherapy. All pretreatment levels were in the normal range, however, decreased by 50% after 1 to 2 courses of therapy and decreased to suboptimal levels (<20 pg/mL) for the remainder of therapy. After the completion of the treatment, levels arose after 3–4 months in 3 of the participants, however remained for a longer period in the participant that received radiation post chemotherapy treatment. Levels of PTH increased 2–3 fold after 1–2 course of treatment and remained high for the remainder of the course of treatment, between 35–40 pg/mL. There was an inverse relationship found between levels of PTH and 1,25-dihydroxyvitamin D.
					Cisplatin, adriamycin, cyclophosphamide, and/or mitomycin. One participant received radiation after chemotherapy was completed.	
			4 participants		**Labs:** 25 hydroxyvitamin-D, 24,25-dihydroxyvitamin D, 1,25-dihydroxyvitamin D, PTH, calcium, phosphorus, potassium, blood urea nitrogen, creatinine, and urinary creatinine clearance at baseline, and 5 days each course of chemotherapy.	
Yavuz *et al.* [31]	Hyperlipidemic	Rosuvastatin	Prospective cohort	Investigate the possible effect of rosuvastatin on vitamin D metabolism	During winter months	There was a significant increase in
					Dose: Rosuvastatin (10–20 mg doses) was used according to the baseline levels of cholesterol and triglycerides, and according to the index of cardiovascular risk.	
			91 participants		Labs: Lipid parameters, 25 hydroxyvitamin-D, 1,25-dihydroxyvitamin D, renal and liver function tests, electrolytes, bone alkaline phosphatase (B-ALP) were obtained at the baseline and after 8 weeks of rosuvastatin treatment.	25-hydroxyvitamin D from 14.0 to 36.3 ng/mL (*p* < 0.001) and 1,25-dihydroxyvitamin D 22.9 to 26.6 pg/mL (*p* = 0.023),
Fakih *et al.* [35]	Colorectal cancer	Various chemotherapy regimens	Retrospective study315 patients	Investigate the vitamin D status in 315 patients with colorectal cancer treated in a single institute.	The first 25-OH vitamin D assay was used as the baseline in patients with multiple 25-OH vitamin D testing. Chemotherapy status was documented in all patients. Colorectal cancer patients were divided into two categories: “no chemotherapy group:” all patients who did not receive any chemotherapy or whose last chemotherapy treatment was at least 3 months prior to 25-OH vitamin D assay.	Patients in the chemotherapy group were 3.2 times more likely to have very low 25-OH vitamin D levels than patients not receiving chemotherapy (*p* < 0.0001).
		43% of patients: irinotecan-based, 39% of patients:			“Chemotherapy group:” all patients whose baseline 25-OH vitamin D level was obtained during chemotherapy treatment or within 3 months after last dose of chemotherapy.	
		oxaliplatin based, 18% of patients: fluoropyrimidine				
Ertugrul *et al.* [32]	Hyperlipidemic	Rosuvastatin	prospective, randomized design	Compare the influences of rosuvastatin and fluvastatin on the levels of 25-hydroxyvitamin D.	During winter months	There was a significant increase in 25-hydroxyvitamin D from 11.8 to 35.2 ng/mL (*p* < 0.001) with rosuvastatin treatment, No significant change in 25-hydroxyvitamin D was observed with fluvastatin treatment (9.6 to 10.2 ng/mL, *p* = 0.557). Rosuvastatin significantly increased 25-hydroxyvitamin D levels compared to fluvastatin (*p* < 0.001) (18.3–24.02 *vs*. 19.4–20.7 ng/mL)
					Dose: rosuvastatin 10 mg (Crestor) or fluvastatin 80 mg XL (Lescol XL) for 8 weeks.	
		Fluvastatin	134 participants were randomized, 1:1		Labs: Lipid parameters, 25 hydroxyvitamin-D, 1,25-dihydroxyvitamin D, renal and liver function tests, electrolytes, bone alkaline phosphatase (B-ALP) were obtained at the baseline and after 8 weeks of treatment.	

**Table A3:** Summary of calcitriol + paclitaxel pharmacokinetics in patients with solid tumors, oral administration [30].

No. of patients	Dose (µg/day)	Dose (µg/week)	T^1^/_2_ (h)	C_max_ (ng/mL)	AUC_0–24_ h(ng h/mL)	CL/F (mL/min)
3	4	28	21 (15–29)	0.21 (0.16–0.29)	2.4 (2.3–3.6)	23 (32–50)
3	6	42	21 (8.7–34)	0.25 (0.23–0.37)	2.4 (2.1–4.0)	50 (29–70)
2	8	56	18 (17–19)	0.27 (0.14–0.41)	3.2 (2.4–4.0)	54 (42–65)
2	11	77	20 (16–24)	0.59 (0.57–0.61)	7.0 (6.9–7.0)	30 (30–31)
3	13	91	13 (5.3–27)	0.37 (0.3–0.9)	3.7 (3.2–6.5)	64 (37–80)
2	17	119	34 (2.5–42)	0.55 (0.39–0.71)	5.9 (4.5–7.4)	57 (41–72)
3	22	154	23 (15–36)	0.46 (0.42–0.54)	5.5 (5.1–6.3)	75 (62–109)
2	29	203	25 (25–26)	0.71 (0.66–0.76)	8.0 (7.7–8.2)	66 (65–67)
6	38	266	25 (15–31)	1.10 (0.32–1.4)	8.1 (5.8–11.0)	91 (62–123)

**Table A4:** Summary of calcitriol with and without dexamethasone, iv administration.

Study	No. of patients	Cancer type	Dose (µg/week)	T^1^/_2_ (h)	C_max_ (ng/mL)	AUC_0–24_ h(ng h/mL)	AUC_0–72_ h (ng h/mL)	Other Drugs
Fakih *et al*. [23]	3	Solid	10	13.5 ± 2.9	0.46 ± 0.21	4.59 ± 0.91		
Fakih *et al*. [23]	3	Solid	15	12.3 ± 0.9	0.77 ± 0.37	5.92 ± 1.00		
Fakih *et al*. [23]	3	Solid	20	12.5 ± 1.9	1.01 ± 0.22	8.32 ± 1.04		
Fakih *et al*. [23]	3	Solid	26	11.6 ± 1.4	1.45 ± 0.47	12.43 ± 3.64		
Fakih *et al*. [23]	3	Solid	34	13.3	1.44 ± 0.84	9.89 ± 3.05		
Fakih *et al*. [23]	3	Solid	44	19.0 ± 1.5	2.72 ± 1.39	17.87 ± 10.72		
Muindi *et al*. [26]	3	Prostate	57	16.3 ± 2.0	4.16 ± 1.78		26.90 ± 5.00	dexamethosone
Fakih *et al*. [23]	3	Solid	57	20.9 ± 3.6	3.80 ± 2.38	24.15 ± 8.62		
Muindi *et al*. [26]	4	Prostate	74	18.6 ± 3.9	4.74 ± 1.13		30.94 ± 6.61	dexamethosone
Fakih *et al*. [23]	3	Solid	74	16.1 ± 4.3	6.68 ± 1.42	35.65 ± 8.01		
Muindi *et al*. [26]	3	Prostate	96	8.7 ± 2.3	10.12 ± 2.17		54.41 ± 15.50	dexamethosone
Fakih *et al*. [23]	3	Solid	96	18.2 ± 1.9	4.23 ± 1.12	25.85 ± 4.41		
Muindi *et al*. [26]	6	Prostate	125	14.6 ± 0.6	11.17 ± 2.62		53.50 ± 10.49	dexamethosone
Muindi *et al*. [26]	4	Prostate	163	11.1 ± 1.7	12.56 ± 1.31		72.22 ± 6.92	dexamethosone
